# The Impact of Medical Face Masks and Rehabilitation Duration on the Performance Output and Outcomes of Cardiologic Rehabilitants

**DOI:** 10.3390/jcm13041086

**Published:** 2024-02-14

**Authors:** Nils Klophaus, Udo F. Wehmeier, Julia Forstner, Armin Jansen, Herbert Probst, Stephan Grüter, Thomas Hilberg

**Affiliations:** 1 Department of Sports Medicine, University of Wuppertal, Moritzstr. 14, 42117 Wuppertal, Germany; nils-klophaus@gmx.de (N.K.); hilberg@uni-wuppertal.de (T.H.); 2 Cardiowell, Arrenberger Str. 20, 42117 Wuppertal, Germany

**Keywords:** cardiological rehabilitation, COVID-19, face mask

## Abstract

During the COVID-19 pandemic, wearing a medical face mask became mandatory in daily life and also in cardiological rehabilitation. In order to investigate whether the performance and outcomes of cardiological rehabilitation were affected by face masks, we compared data from patients who underwent rehabilitation with face masks with data from patients without face masks. In total, 114 patients from an ambulant rehabilitation center were included. Of them, 60 patients completed rehabilitation without a face mask (NFM). In contrast, 54 patients (with a face mask, WFM) completed their rehabilitation during the COVID-19 pandemic and had to wear medical face masks for the entire day and also during ergometer training or other interventions. Subgroups were formed with patients who accepted to extend rehabilitation for one week (4 WG); the other patients only completed 3-week rehabilitation (3 WG). We analyzed the performance and outcomes of all groups (NFM; WFM, 3 WG and 4 WG). At baseline, no group differences were detected. All groups significantly improved their power output and heart rate recovery, without any group differences. We conclude that face masks and also an additional rehabilitation week do not affect the exercise performance or outcomes of out-house cardiological rehabilitation.

## 1. Introduction

During the SARS-CoV-2 (COVID-19) pandemic, the use of face masks was recommended to prevent contracting and transmitting COVID-19 [1,2]. Some studies have investigated the effects of face masks in acute exercise and for healthy people, and either no or only minor effects have been reported [3,4,5,6,7,8]. Also, during high-intensity resistance training in elite athletes, no negative mask effects were detected [9]. However, for sedentary people, Umutlu et al. [10] reported negative impacts during aerobic exercise with masks, while for end-stage lung disease patients, no negative mask effects were found during a 6 min walking test [11]. For cardiac patients and patients completing rehabilitation, the effects of face masks have still not been investigated.

In Germany, patients have the right to in- or outpatient rehabilitation subsequent to myocardial infarction, percutaneous coronary intervention (PCI), heart surgery, or other severe cardiovascular diseases [12]. Rehabilitation lasts for three weeks but can be extended for one week. According to international guidelines [13], patients train according to the standard method for the rehabilitation of cardiac patients [12], in most cases on bicycle ergometers. Ergometer training is complemented by walking, calisthenics, and also seminars focusing on lifestyle and diet.

In this study, we wanted to investigate whether (1) wearing a face mask has an impact on outcomes in German outpatient cardiological rehabilitation and (2) whether the rehabilitation duration (3 or 4 weeks) affects the outcome in outpatient cardiological rehabilitation.

In order to answer the question as to whether face masks have effects on general performance diagnostic parameters, which are usually measured during cardiological rehabilitation, or the outcomes of rehabilitation at all, we compared the data of patients who completed rehabilitation without masks with those of patients who had to wear face masks during their rehabilitation. Furthermore, in subgroup analysis, we examined if an additional rehabilitation week improved the outcomes significantly by comparing the data of patients who underwent 3-week out-house rehabilitation with those of patients who underwent 4-week out-house rehabilitation.

## 2. Materials and Methods

### 2.1. Patients and Patient Recruitment

Participants were recruited from those referred from the Heart Center Wuppertal to the rehabilitation center directly after an event like myocardial infarction and/or a percutaneous coronary intervention (PCI). The patients started their rehabilitation program 2–4 weeks after their stationary stay in the Heart Center. The first recruitment period for patients without face masks (NFM groups) started in October 2019 and ended in March 2020, and the second recruitment period for patients with face masks (WFM groups) was from January to June 2022. Patients who fulfilled the inclusion criteria (age 30–70 years, no defibrillator or cardiac pacemaker, ejection fraction > 40, no ischemic symptoms or other ECG abnormalities, no orthopedic restrictions, no signs of depression, being able to perform bicycle ergometer training) were asked to participate. In order to reflect the normal distribution of the various cardiovascular diseases of a rehabilitation center, no further exclusion markers were defined. Participants performed their normal rehabilitation without any changes in the intervention. They had to agree that their data could be used for this study. Three patients of the WFM group and five of the NFM group dropped out as they could not train regularly for medical reasons due to acute infections or were not able to pass the post-test for the same reasons.

### 2.2. Patient Groups and General Intervention Schedule

For cardiovascular diseases, the standard out-house rehabilitation in Germany lasts for three weeks [12]. In agreement with the supervising cardiologist during rehabilitation, the patients normally receive the offer to extend the rehabilitation for one week and the patients can decide whether to accept this offer or not. Patients who accepted the offer then belonged to the 4-week group (4 WG), and all other patients to the 3-week group (3 WG). The aim of this study was to include 30 patients in every group (NFM, 3 weeks; NFM 4 weeks; WFM 3 weeks; WFM 4 weeks). The necessary sample size was calculated according to a previous study [14] with a power of 0.8 using an expected gain in the peak power output of 10 W and expected group difference of 5 Watts.

On the first day of their rehabilitation, the patients were tested with a standardized incoming ergometric examination (pre-test), which was conducted according to a recommended standard protocol for cardiac patients (2 min intervals, increase 25 W/interval) [15]. The patients then underwent a rehabilitation comprising 3–4 standardized moderate continuous endurance training units per week on bicycle ergometers (EC 3000, Custo-med, Ottobrunn, Germany) as well as supervised walking sessions and calisthenics (for details, see Section 2.3). All interventions were adjusted to a rate of perceived exertion (RPE) 13 on the Borg RPE scale [16]. On the last intervention day, the outgoing ergometric examination (post-test) was performed exactly in the same way as the pre-test. All patients received their necessary medical therapy (thrombocyte aggregation blocker, ACE blocker, blood lipid reducer, beta blocker, or antihypertension drugs) when entering the rehabilitation. The medication was optimized during the rehabilitation by the same physician. For the medication, no significant group differences were observed.

### 2.3. Rehabilitation at the Cardiowell Rehabilitation Center

Our study was executed in cooperation with the out-house rehabilitation center “Cardiowell, Center for Prevention and Rehabilitation” in Wuppertal, Germany, which is situated at the Heart Center Wuppertal. The rehabilitation at Cardiowell is guided by cardiologists and the training sessions are supervised by sport therapists. During the out-house rehabilitation, the patients undertook their interventions from Monday to Friday, while the weekend was free. The typical rehabilitation program provided daily training sessions lasting 25–35 min, including a warm-up (7 min) and cool-down phase (3 min) on bicycle ergometers, applying a moderate continuous protocol at 65–70% of the maximum heart rate. The training was accompanied by daily supervised walking, supervised calisthenics in groups, and lectures on lifestyle, behavior, and risk factors. 

### 2.4. Registration and Ethics

The rehabilitation in Germany is financed either by the German pension insurance or health insurance, who both gave written consent for this study. This study was approved by the Ethic Committee of the University Wuppertal (MS/BB 180824 from 16 September 2019 for patients without face masks; MS/AE 220118 from 14 February 2022 for patients with face masks) in compliance with the Declaration of Helsinki.

### 2.5. Performance Parameters

At the beginning and the end of their rehabilitation, the patients were examined as described (Section 2.2). The following parameters were monitored during these examinations: heart rate at rest (bpm; beats per minute), maximum heart rate (bpm), heart rate recovery after 2 min (bpm), blood pressure at rest (mmHG), maximum blood pressure (mmHG), peak power output (W), relative peak power output (Wattrel; W/kg bodyweight).

### 2.6. Data Analysis

The statistical analyses were conducted using the SPSS program version 28 (SPSS Inc., Chicago, IL, USA). Kolmogorov–Smirnov test was used to test for a normal distribution. As the distribution differed between the parameters, we decided to calculate all data as normally distributed. Differences in baseline data and anthropometric data were analyzed with unpaired *t*-tests, and differences for each group were analyzed with paired *t*-tests. A multifactor ANOVA with repeated measures (MANOVA) was performed to analyze the influences of the time points and the groups on paired samples and to compare the groups in the pre- and post-tests. For all tests, the significance level was set at *p* ≤ 0.05.

## 3. Results

### 3.1. Comparison of Results with and without Face Masks

The anthropometric data in Table 1 exhibit significant group differences only in the weight between the 4-week groups (*p* = 0.035). No further group differences were found. Furthermore, no differences were found for the three most relevant cardiological diseases, which were the reason for the rehabilitation (Table 1).

When comparing the numbers of training sessions for the patient groups (Table 1), the participants in the WFM groups had significantly more walking sessions (3 weeks: NFM: 4 sessions; WFM: 5 sessions; *p* = 0.003; 4 weeks: NFM: 5 sessions; WFM: 9 sessions; *p* ≤ 0.001). The number of calisthenics sessions differed significantly only between the 4-week groups (NFM: 10 sessions; WFM: 11 sessions; *p* = 0.048).

#### Outcomes with and without Face Masks

At baseline, no differences in the tested parameters were detected between the WFM and NFM groups (Table 2). At the end of the rehabilitation, all groups had significantly improved their maximum power output and also their relative power output (NFM: 3 weeks: +16 W, *p* ≤ 0.001, +0.2 W/kg, *p* ≤ 0.001; 4 weeks: +20 W, *p* ≤ 0.001, +0.2 W/kg, *p* ≤ 0.001; WFM: 3 weeks: +17 W, *p* ≤ 0.001, +0.2 W/kg, *p* ≤ 0.001; 4 weeks: +26 W, *p* ≤ 0.001, +0.3 W/kg, *p* = 0.001) without group differences (Table 2). The heart rate recovery after 2 min improved significantly only in the WFM groups (3 weeks: +4.8 bpm, *p* = 0.003; 4 weeks: +6.7 bpm, *p* = 0.003) without group differences. Blood pressure at rest was only significantly affected in the 3-week NFM group (systolic) and the 3-week WFM group (systolic and diastolic).

### 3.2. Comparison of 3-Week vs. 4-Week Rehabilitation

As no relevant parameter was significantly affected by face masks (Table 2), outcome and training comparisons of the 3- and the 4-week groups were conducted without NFM or WFM subgroups, and those for the combined 3- and 4-week groups included WFM and NFM patients. The anthropometric data of these combined groups (Table 3) exhibited no significant differences. Due to an extended rehabilitation, the 4-week group had significantly more ergometer training sessions, more calisthenics sessions, and also more walking sessions (Table 3).

#### Outcomes of 3-Week vs. 4-Week Rehabilitation

In pre-tests, no significant differences between the combined 3- and 4-week groups were found. At the end of the rehabilitation, both groups had significantly improved the maximum and relative power output, but again, MANOVA revealed no group differences. In post-tests, the 3-week group had a significantly reduced BPdia in rest, while the 4-week group reached a significantly higher maximal heart rate. However, significant group differences were not detected either for blood pressure or heart rate recovery (Table 4).

## 4. Discussion

The two main goals of this study were to evaluate the impact of face masks on the maximum performance of cardiac patients and on the outcomes of out-house German cardiac rehabilitation, and to determine whether an extension of the standard 3-week rehabilitation by an additional week can improve the rehabilitation outcomes.

### 4.1. Impact of Face Masks

The maximum performance parameter tests and the improvements in the tested parameters did not reveal any differences between the WFM and NFM groups. This indicates that face masks had no negative effects for cardiac patients during acute and exhausting exercises. This observation is in accordance with other studies that investigated the effect of face masks during acute exercise in healthy people and found no or only minor effects [3,5,6,7,8,17]. In these studies, some discrete changes in capillary pCO2 and pO2 within the physiologic range and increasing RPEs were reported [8] as well as small shifts of the ventilatory anaerobic threshold [6]. Although no significant differences became obvious, the percentage improvements in the absolute and relative power outputs in this study were slightly greater in WFM (peak power output: WFM: +21 W, +19%; NFM: +18 W, +16%; relative power output: WFM: +0.3 W/kg, +19%, NFM: +0.2 W/kg, +16%). This may hint that the masks could have forced some discrete changes in the ventilatory parameters by causing hypoxic conditions, which can improve adaptation processes to training, including the respiratory muscles [18]. The training in our study was controlled by the RPE but the fact that all groups improved quite similarly indicates that wearing a face mask had no great effect on the RPE and performance intensities during the training sessions. As our patients were not examined by spiroergometry, ventilatory parameters were not available. The fact that the maximal performance of the patients was not affected by face masks indicates that ventilatory parameters were not impaired by masks. This observation is in good accordance with the review of Barbeito-Caamaño [19], who also found no differences for exercise testing in patients with masks. Also, the maximum heart rates and the blood pressure were not affected by the rehabilitation in either group. As all patients received their necessary medication to stabilize these parameters, one can assume that the medication was the major effector of these parameters and not the improvement in the physical fitness. Similar results were reported in other studies with different patient groups [20,21,22,23,24], although all of these studies lasted longer than 4 weeks. Measurable effects of a training program on the blood pressure may only become apparent if the use of hypertensive drugs is terminated before the training program, as shown for hypertensive patients [25]. Since no abnormalities as a result of wearing face masks during the maximum performance tests were observed, one can conclude that exercise and stress tests can be performed with cardiac patients including those wearing face masks safely, without significant differences in the results.

One of the main goals of cardiac rehabilitation is to obtain or improve functional capacities [13]. This goal was not affected by face masks in our study, with us finding similar improvements for all tested markers in all groups. As patients in the WFM groups had to wear masks for the whole day, during all interventions, and also during the peak performance tests, we conclude that the overall impact of masks is relatively small and does not influence the rehabilitation outcome at all. Furthermore, the fact that the improvements in the peak power output in all groups were in the same range as in a previous study [14] at the same rehabilitation center indicates that the overall outcome of standard rehabilitation depends mainly on the efficiency of the rehabilitation training program.

### 4.2. 3-Week vs. 4-Week Rehabilitation

In our results, significant differences between the combined 3- and 4-week groups were found only in the number of training sessions, which was expected. However, that the increased number of training sessions did not lead to better improvements in any of the tested parameters was not expected. As we compared only the final outcomes, it is not clear if the members of the 4-week group had reached the same performance level already after 3 weeks or if they still improved their performance parameters during the last week. However, that the additional week had no significant effects on the improvements suggests that the training management in the rehabilitation can be improved. Either the patients need more intense or better individualized training in the second half of their rehabilitation, as they have already improved their performance levels after the first 1–2 weeks, or the daily training sessions cause them too much exhaustion, which limits further improvements. Behrens et al. [26] have shown that an individualized training program with reduced training sessions for cardiac patients can be beneficial when compared to a daily standard program. In this regard, our data show that standard 3- or 4-week rehabilitation programs with or without face masks improve the fitness of cardiological patients equally, suggesting that upgrades to the rehabilitation training to gain even better improvements are still possible.

From our results, we can conclude that wearing face masks does not significantly affect the performance (maximum power output) of cardiac patients. Also, the outcomes of a 3-week or 4-week out-house rehabilitation program were not affected by wearing face masks over whole days and during all interventions. This shows that cardiac rehabilitation can be performed at normal intensities in pandemic phases requiring face masks. The training programs during cardiac rehabilitation, however, can still be improved as differences between 3- and 4-week rehabilitations were not observed, independent of face masks.

### 4.3. Limitations

This study was performed as a single-center study. As the decision for the optional 4th week is made within the first 3 weeks of the rehabilitation program, we were not able to randomize the patients before they entered the study. As two-thirds of the patients during the first recruitment period in the rehabilitation center accepted the extension by 1 week, the recruitment of the 4-week group was completed from October to the first January week, while 15 patients (50%) of the 3-week group were recruited between January and in the third week of March. During the second recruitment period, the 30 participants for the 3-week group were reached first. Therefore, seasonal effects, e.g., affecting daily activities, cannot be completely excluded. Furthermore, the recruitment of the patients with face masks happened during the COVID-19 pandemic. As such, restrictions in everyday life, like closed gyms and reduced sports offerings, could have impacted the general fitness and the daily range of motion of the participants and their physical performance.

One more limitation was the fact that participants of the face mask group were allowed to decide themselves which face mask (surgical mask or filtering face piece type 2) they wore, and they also could change the mask type within or between days.

## 5. Conclusions

Wearing a face mask for the whole day during out-house cardiac rehabilitation had no negative effect on peak performance parameters and on the outcomes of a 3- or 4-week rehabilitation. Between a 3- and a 4-week rehabilitation, outcome differences were observed only for the HR at rest at the post-test, probably due to optimized medication, while no further outcome differences were detected.

## Figures and Tables

**Table 1:** Anthropometric data and training volumes of the participants (3 (3 WG)- and 4 (4 WG)-week groups, without (NFM) and with (WFM) mask) during their rehabilitation. Values are given as mean ± SD. BMI, body mass index; ^#^, ICD-10 classification; AMI, acute transmural myocardial infarct; PTCI, presence of an implant or transplant after coronary vascularplasty; valvular aortic stenosis; *p* = *t*-test. Significant differences between the groups are marked: * *p* ≤ 0.05, ** *p* ≤ 0.01, *** *p* ≤ 0.001.

Parameter	NFM (*n* = 57) 3 Weeks (*n* = 29) 4 Weeks (*n* = 28)	WFM (*n* = 49) 3 Weeks (*n* = 27) 4 Weeks (*n* = 22)	P (NFM vs. WFM)
Sex			
Male *n* (%)			
3 weeks	25 (86.2%)	21 (77.8%)	
4 weeks	25 (89.3%)	19 (86.4%)	
Female *n* (%)			
3 weeks	4 (13.8%)	6 (22.2%)	
4 weeks	3 (10.7%)	3 (13.6%)	
Age (Years)			
3 weeks	57.5 ± 6.6	57.6 ± 8.2	0.971
4 weeks	55.6 ± 7.5	56.4 ± 6.8	0.697
Height (cm)			
3 weeks	177.5 ± 8.1	176.2 ± 10.7	0.629
4 weeks	178.6 ± 10.3	176.7 ± 9.0	0.498
Weight (kg)			
3 weeks	87.1 ± 13.4	85.7 ± 16.2	0.735
4 weeks	93.3 ± 13.8	84.9 ± 13.5	0.035 *
BMI (kg/m^2^)			
3 weeks	27.7 ± 4.1	27.5 ± 4.8	0.909
4 weeks	29.3 ± 4.0	27.1 ± 3.6	0.054
Ejection fraction (%)			
3 weeks	61.2 ± 6.3	57.6 ± 9.1	0.093
4 weeks	58.2 ± 8.3	58.3 ± 8.8	0.973
AMI (I210–213) ^#^			
3 weeks	20 (68.9%)	20 (74.1%)	0.550
4 weeks	18 (64.3%)	17 (77,2%)	0.548
PITC (Z951) ^#^			
3 weeks	19 (65.5%)	22 (81.5%)	0.133
4 weeks	20 (71.4%)	19 (86.4%)	0.457
VAS (I351)			
3 weeks	4 (13.8%)	3 (11.1%)	0.810
4 weeks	3 (10.7%)	2 (9.1%)	0.131
Ergometer training			
sessions (*n*)			
3 weeks	9.8 ± 0.7	9.8 ± 0.6	0.941
4 weeks	14.7 ± 0.5	14.6 ± 0.7	0.566
Calisthenics sessions (*n*)			
3 weeks	6.9 ± 0.9	7.4 ± 2.0	0.190
4 weeks	10.0 ± 1.0	10.8 ± 1.7	0.048 *
Walking sessions (*n*)			
3 weeks	3.7 ± 1.5	5.2 ± 2.2	0.003 **
4 weeks	5.4 ± 2.4	8.6 ± 2.9	<0.001 ***

**Table 2:** Comparison of the maximum performance parameters of the participants (3 (3 WG)- and 4 (4 WG)-week groups, without (NFM) and with (WFM) face mask) at the beginning (pre) and the end (post) of the rehabilitation. Values are given as mean ± SD. HR, heart rate; BP, blood pressure. Significant group changes (paired *t*-test) from pre to post are marked: * *p* ≤ 0.05, ** *p* ≤ 0.01, *** *p* ≤ 0.001. MANOVA was used for group comparisons (NFM vs. WFM).

Parameter	NFM (*n* = 57)		WFM (*n* = 49)		MANOVA (NFM vs. WFM)
	3 Weeks (*n* = 29)		3 Weeks (*n* = 27)		
	4 Weeks (*n* = 28)		4 Weeks (*n* = 22)		
	Pre	Post	Pre	Post	
Watt_max_ (W)					
3 weeks	109.5 ± 29.0	125.8 ± 27.3 ***	103.7 ± 40.1	120.8 ± 47.0 ***	0.874
4 weeks	114.4 ± 34.3	133.6 ± 43.2 ***	119.3 ± 46.1	145.3 ± 51.8 ***	0.190
Watt_rel_ (W/kg)					
3 weeks	1.26 ± 0.26	1.46 ± 0.29 ***	1.21 ± 0.43	1.41 ± 0.49 ***	0.995
4 weeks	1.23 ± 0.35	1.44 ± 0.44 ***	1.40 ± 0.47	1.70 ± 0.55 ***	0.088
HR_rest_ (bpm)					
3 weeks	74.7 ± 13.7	76.1 ± 8.5	79.0 ± 11.8	79.2 ± 11.2	0.723
4 weeks	75.5 ± 14.1	73.4 ± 10.0	72.2 ± 13.5	68.7 ± 6.6	0.701
HR_max_ (bpm)					
3 weeks	116.7 ± 21.0	120.8 ± 19.5	117.7 ± 22.1	119.0 ± 23.4	0.396
4 weeks	115.0 ± 18.2	119.5 ± 21.6	120.1 ± 23.4	127.0 ± 23.3 *	0.469
HR recovery (bpm)					
3 weeks	30.8 ± 10.3	33.9 ± 11.7	28.5 ± 13.3	33.3 ± 15.0 **	0.405
4 weeks	29.7 ± 10.9	38.0 ± 24.1	31.1 ± 12.6	37.8 ± 14.0 **	0.731
BP_sys rest_ (mmHG)					
3 weeks	122.0 ± 15.3	112.8 ± 20.7 **	116.9 ± 23.2	109.3 ± 14.7 *	0.704
4 weeks	119.8 ± 21.8	114.0 ± 14.6	120.5 ± 23.4	113.3 ± 17.1	0.833
BP_dia rest_ (mmHG)					
3 weeks	72.6 ± 10.0	68.7 ± 9.9	73.8 ± 11.9	67.8 ± 10.1 *	0.505
4 weeks	72.8 ± 11.1	70.7 ± 10.8	70.8 ± 15.1	69.1 ± 11.5	0.907
BP_sys max_ (mmHg)					
3 weeks	178.4 ± 33.7	177.6 ± 21.4	172.0 ± 39.5	165.0 ± 34.2	0.500
4 weeks	165.3 ± 29.5	166.6 ± 23.9	176.4 ± 39.1	186.1 ± 37.1	0.309
BP_dia max_ (mmHg)					
3 weeks	90.5 ± 16.0	89.4 ± 16.9	84.8 ± 13.9	83.4 ± 15.2	0.948
4 weeks	89.4 ± 13.3	88.0 ± 14.0	89.1 ± 19.7	89.9 ± 18.4	0.667

**Table 3:** Anthropometric data and training volumes of the participants (3- and 4-week groups) during their rehabilitation. Values are given as mean ± SD. BMI, body mass index; ^#^, ICD-10 classification; AMI, acute transmural myocardial infarct; PTCI, presence of an implant or transplant after coronary vascularplasty; VAS, valvular aortic stenosis. Significant differences (unpaired *t*-test) between the groups are marked: *** *p* ≤ 0.001.

Parameter	3 Weeks (*n* = 56)	4 Weeks (*n* = 50)	*p*
Sex			
Male, *n* (%)	46 (82.1)	44 (88.0)	
Female, *n* (%)	10 (17.9)	6 (12.0)	
Age (Years)	57.5 ± 7.3	56.0 ± 7.1	0.271
Height (cm)	176.9 ± 9.4	177.7 ± 9.7	0.634
Weight (kg)	86.4 ± 14.7	89.6 ± 14.1	0.258
BMI (kg/m^2^)	27.6 ± 4.4	28.3 ± 3.9	0.361
Ejection fraction (%)	59.5 ± 7.9	58.3 ± 8.4	0.445
AMI (I210–213) ^#^	40 (68.9%)	35 (70.0%)	0.233
PTCI (Z951) ^#^	41 (71.4%)	39 (78.0%)	0.453
VAS (I351) ^#^	7 (12.5%)	5 (10.0%)	0.279
Ergometer training sessions (*n*)	9.8 ± 0.6	14.7 ± 0.6	<0.001 ***
Calisthenics sessions (*n*)	7.1 ± 1.5	10.4 ± 1.4	<0.001 ***
Walking sessions (*n*)	4.4 ± 2.0	6.8 ± 3.1	<0.001 ***

**Table 4:** Comparison of the maximum performance parameters of the participants (3- and 4-week groups) at the beginning (pre) and the end (post) of the rehabilitation. HR, heart rate; BP, blood pressure. Values are given as mean ± SD. Significant differences between the pre- and the post-test within the groups (paired *t*-test) are marked: * *p* ≤ 0.05, ** *p* ≤ 0.01, *** *p* ≤ 0.001. MANOVA was used for group comparisons (3-week vs. 4-week).

Parameter	3 Weeks (*n* = 56)		4 Weeks (*n* = 50)		MANOVA
	Pre	Post	Pre	Post	
Watt_max_ (W)	106.7 ± 34.6	123.4 ± 37.8 ***	116.5 ± 39.6	138.8 ± 47.0 ***	0.109
Watt_rel_ (W/kg)	1.24 ± 0.35	1.43 ± 0.40 ***	1.30 ± 0.41	1.56 ± 0.50 ***	0.177
HR_rest_ (bpm)	76.7 ± 12.9	77.6 ± 9.9	74.1 ± 13.8	71.3 ± 8.9	0.153
HR_max_ (bpm)	117.2 ± 21.3	120.0 ± 21.3	117.3 ± 20.6	122.8 ± 22.4 *	0.307
HR recovery (bpm)	29.7 ± 11.8	33.6 ± 13.3 ***	30.3 ± 11.6	37.9 ± 20.1 **	0.161
BP_sys rest_ (mmHG)	119.5 ± 19.5	111.1 ± 18.0 ***	120.1 ± 22.3	113.7 ± 15.6 *	0.591
BP_dia rest_ (mmHG)	73.2 ± 10.9	68.3 ± 9.9 **	71.9 ± 12.9	70.0 ± 11.0	0.208
BP_sys max_ (mmHg)	175.3 ± 36.4	171.6 ± 33.1	170.2 ± 34.1	175.2 ± 31.6	0.158
BP_dia max_ (mmHg)	87.8 ± 15.2	86.5 ± 16.2	89.3 ± 16.3	88.8 ± 16.0	0.801

## Data Availability

Due to privacy restrictions, all data are stored at the researchers’ institution. Qualified researchers will be able to gain access via application to the corresponding author.
